# Memory-Enhancing Effects of Mangosteen Pericarp Water Extract through Antioxidative Neuroprotection and Anti-Apoptotic Action

**DOI:** 10.3390/antiox10010034

**Published:** 2020-12-30

**Authors:** Yeonsoo Oh, Ha Thi Thu Do, Sunyoung Kim, Young-Mi Kim, Young-Won Chin, Jungsook Cho

**Affiliations:** 1 College of Pharmacy and Integrated Research Institute for Drug Development, Dongguk University-Seoul, Dongguk-ro 32, Ilsandong-gu, Goyang, Gyeonggi 10326, Korea; dhdustn92@naver.com (Y.O.); doha201191@gmail.com (H.T.T.D.); chupy027979@naver.com (S.K.); 2 College of Pharmacy, Seoul National University, Seoul 08826, Korea; 0210121@hanmail.net (Y.-M.K.); ywchin@snu.ac.kr (Y.-W.C.)

**Keywords:** mangosteen pericarp, mangosteen pericarp water extract, antioxidant, neuroprotection, anti-apoptosis, β-secretase, acetylcholinesterase, cognitive dysfunction, Alzheimer’s disease

## Abstract

Mangosteen has long been utilized as a traditional medicine in Southeast Asia. Diverse extracts of mangosteen pericarp and its bioactive xanthones exhibit various bioactivities. However, the pharmacological potential of mangosteen pericarp water extract (MPW) has not been reported yet. This study used primary cultured rat cortical cells to investigate the effect of MPW on neurotoxicity. We found that MPW inhibited neurotoxicity and production of reactive oxygen species triggered by Aβ_(25–35)_ or excitatory amino acids. MPW inhibited caspase 3 activation and DNA fragmentation in Aβ_(25–35)_- or N-methyl-D-aspartate-treated cells, suggesting an anti-apoptotic action. Additionally, MPW reduced lipid peroxidation and scavenged 1,1-diphenyl-2-picrylhydrazyl radicals, assuring its antioxidant property. Furthermore, MPW suppressed β-secretase and acetylcholinesterase activities. These findings prompted us to evaluate its effect on memory dysfunction in scopolamine-treated mice using Morris water maze test. Oral administration of MPW at the dosage of 50, 100, or 300 mg/kg for four days significantly decreased the latency time to find the platform and markedly increased the swimming time in the target quadrant. Taken together, our results suggest that MPW exerts memory-enhancing effect through antioxidative neuroprotection and anti-apoptotic action. Accordingly, MPW may have a potential to prevent or treat memory impairment associated with Alzheimer’s disease.

## 1. Introduction

Alzheimer’s disease (AD) is a chronic neurodegenerative disorder accompanied by progressive memory and cognitive impairment. The pathological features of AD include the extracellular accumulation of senile plaques and the intracellular aggregation of neurofibrillary tangles. Senile plaques are mainly composed of aggregated β-amyloid (Aβ) generated from amyloid precursor protein (APP) upon enzymatic cleavage by β- and γ-secretases [1,2]. Aβ is thought to play an important role in neurotoxicity both in vitro and in vivo. Aβ peptide causes destabilization of calcium homeostasis and induction of apoptosis, ultimately leading to neurotoxicity [3]. Accordingly, the Aβ pathway has been one of the promising targets for the development of putative disease-modifying drugs to treat AD. Various approaches to combat the Aβ pathway, including inhibitors of β- or γ-secretase, or anti-Aβ monoclonal antibodies, have been challenged. However, no drug has been introduced into the clinic yet [4]. Furthermore, Aβ peptide is considered to induce oxidative damage in neuronal cells by generating reactive oxygen species (ROS) [5]. Thus, oxidative stress either triggered by Aβ peptide or any other stimuli may be one of the critical causative factors in AD. Based on these findings, antioxidants that alleviate ROS and mitigate oxidative stress-induced neuronal cell death have also been proposed as an intriguing approach to prevent or treat AD [6]. Unfortunately, however, the results from clinical studies have been disappointing to date. Considering the multifactorial and heterogeneous characteristics of the pathophysiology of AD, various approaches have been attempted to explore the favorable efficacies of antioxidant therapies in AD [7,8].

Glutamate is the most abundant excitatory amino acid neurotransmitter in the central nervous system (CNS). Glutamate transmission is mediated by ionotropic receptors, such as N-methyl-D-aspartic acid (NMDA), α-amino-3-hydroxy-5-methylisoxazole-4-propionic acid, and kainate receptors, as well as by metabotropic (G protein-coupled) receptors [9]. Although it is well documented that glutamate transmission is involved in synaptic plasticity and cognitive functions in the brain [10], an excessive quantity of glutamate release is also known to be involved in many acute and chronic neuropathological conditions, including cerebral ischemia, epilepsy, and AD [11]. Accumulating evidence indicates that excess glutamate overactivates NMDA receptors and causes a redundant influx of calcium ions into cells and excessively generates ROS, which can eventually trigger apoptotic or necrotic neuronal cell death, resulting in excitotoxicity [12,13]. Thus, targeting glutamate transmission to prevent excitotoxicity, specifically by modulating NMDA receptor function, has been an attractive approach for the development of pharmacological interventions to treat neurological disorders such as AD. Memantine, a non-competitive NMDA receptor antagonist, is a well-known drug clinically used for the treatment of AD [14].

Mangosteen (*Garcinia mangostana* L.) is a tropical tree cultivated in Southeast Asia. The seeds and pericarps of the fruit have been traditionally used for centuries to treat skin infections, wounds, and dysentery [15,16,17]. Additionally, mangosteen products containing pulp and pericarps are sold worldwide, including in the U.S. market as nutritional supplements in the form of beverages or tablets [15,18,19]. The mangosteen pericarp has been reported to contain at least 50 different bioactive compounds, such as xanthones, polyphenols, and catechins [16,20]. Compared to the edible aril parts of the fruit, the pericarp consists of 10 times more phenolic compounds and 20 times more antioxidant activities [19].

Diverse extracts prepared from mangosteen pericarp through different methods have been reported to exhibit a wide variety of biological activities, including antioxidant, anti-inflammatory, analgesic, anti-cancer, and neuroprotective effects [15,20]. The methanol extract and chloroform fraction of mangosteen pericarp inhibit the proliferation of human hepatocellular, breast, and colorectal cancer cells and exhibit antioxidant activities [21,22]. Another study has reported that a 50% ethanol mangosteen pericarp extract (MPE) shows antioxidative and neuroprotective activities against H_2_O_2_-induced oxidative stress in NG108-15 neuroblastoma cells [23]. Various xanthone derivatives, including α- and γ-mangostins have been found in mangosteen pericarp and exhibit many of these biological activities [16]. We have recently demonstrated that γ-mangostin, not α-mangostin, isolated from mangosteen pericarp inhibits H_2_O_2_-induced oxidative neurotoxicity in the primary culture of rat cortical cells and reverses scopolamine-induced memory impairment in mice [24]. However, no reports are currently available on the effects of the mangosteen pericarp water extract (MPW) prepared by simplified and easily applicable processes without using organic solvents. Therefore, to elucidate the pharmacological potential of MPW, the present study explored the antioxidative and neuroprotective effects and memory-enhancing properties of MPW using primary cultures of rat cortical cells and scopolamine-treated mice, respectively.

We first investigated the effects of MPW on neuronal cell damage, ROS production, and apoptotic processes triggered by Aβ or excitatory amino acids in the cultured cortical cells. The antioxidative property of MPW was further evaluated by cell-free bioassays, measuring the levels of lipid peroxidation (LPO) and 1,1-diphenyl-2-picrylhydrazyl (DPPH) radical formation in the presence of MPW. Moreover, we examined the impact of MPW on the activities of β-secretase as well as acetylcholinesterase. Finally, we used the Morris water maze (MWM) test to assess the effect of orally administered MPW on the scopolamine-induced memory dysfunction in mice.

## 2. Materials and Methods

### 2.1. Materials

Minimum essential medium (MEM, supplemented with Earle’s salt), fetal bovine serum (FBS), horse serum (HS), antibiotic-antimycotic agent, and β-secretase fluorescence resonance energy transfer (FRET) assay kit were purchased from Invitrogen (Carlsbad, CA, USA). DeadEnd^TM^ Colorimetric terminal deoxynucleotidyl transferase-mediated deoxyuridine triphosphate nick-end labeling (TUNEL) assay kit was obtained from Promega (Madison, WI, USA). Poly-L-lysine, laminin, cytosine arabinoside, DPPH, 3-(4,5-dimethylthiazol-2-yl)-2,5-diphenyltetrazolium bromide (MTT), L-glutamic acid, glucose, L-glutamine, 2′,7′-dichlorofluorescin diacetate (DCFH-DA), trichloroacetic acid, 2-thiobarbituric acid (TBA), acetylthiocholine iodide, 5,5′-dithiobis(2-nitrobenzoic acid) (DTNB), ethylenediaminetetraacetic acid (EDTA), scopolamine, donepezil, and anti-β-actin antibody (monoclonal) were from Sigma-Aldrich (St. Louis, MO, USA). Anti-caspase 3 antibody (8G10), horseradish peroxidase (HRP)-conjugated anti-rabbit immunoglobulin G (IgG), and anti-mouse IgG antibodies were procured from Cell Signaling Technology (Danvers, MA, USA). NMDA was provided by Tocris Bioscience (Bristol, UK) and Aβ_(25–35)_ was from Bachem Ltd. (St. Helens, UK). All other chemicals were of analytical grade.

### 2.2. Animals

Timed-pregnant Sprague-Dawley (SD) rats and ICR mice were purchased from Daehan Biolink (Chungbuk, Korea). Animals were maintained in the animal facility with controlled temperature (22 ± 2 °C), relative humidity (40–60%), and a 12-h light/ 12-h dark cycle. Animals were provided with a standard chow diet and water ad libitum. All the experimental steps, including the care, handling, and use of animals, were performed following the international guidelines (Guide for the Care and Use of Laboratory Animals, Institute of Laboratory Animal Resources, Commission on Life Sciences, National Research Council; National Academy Press: Washington DC, 1996). Prior to the study, the rationale, design, and protocols of the animal experiments were approved by the Institutional Animal Ethical Committee of Dongguk University (Approval Number: IACUC-2016-035-2).

### 2.3. Preparation and Analysis of MPW

The dried pericarps of *Garcinia mangostana* L. (7.9 kg) were obtained from Indonesia and refluxed with distilled water (7.9 L) for 2 h at 100 ℃, yielding the crude extract of MPW (298.7 g). Chromatographic separation of the analytes was performed using the Acquity UPLC system with a BEHC18 column (2.1 × 50 mm, 1.7 μm, Waters, Milford, MA, USA). An aliquot of the sample (2 μL) was injected into the UPLC system for analysis. The mobile phase consisted of 0.1% formic acid in water for solution A and in acetonitrile for solution B. The mobile phase consisting of (A) and (B) was delivered at a flow rate of 0.4 mL/min by the following programmed gradient elution: 30% isocratic for 3 min, 30→70% (B, *v*/*v*) in 4 min, 70% isocratic for 2 min, 70→100% (B) in 2 min, 100% (B) isocratic for 2 min, 100→30% (B) in 0.5 min, and 5% (B) isocratic for 0.5 min for reconditioning post-run. The column temperature was maintained at 40 ℃.

Mass spectrometry was performed on the Xevo G2 Q-TOF MS (Waters, Milford, MA, USA) equipped with an electronic spray ion source under the positive-ion mode. The capillary and cone voltages were 2.7 kV and 15 V, respectively. Nitrogen, used as the cone, and desolvation gas were set at 0 and 600 L/h, respectively. The temperatures of the source and desolvation were 100 and 250 ℃, respectively. Mass spectra were recorded in the range of m/z 50–1200. Spectroscopic data, including UPLC-UV and mass chromatogram, for MPW are depicted in the Supplementary Materials.

### 2.4. Primary Cultures of Rat Cerebrocortical Cells

Primary cultures of rat cerebrocortical cells were carried out as previously described [25,26]. In brief, cerebral cortices were dissected from timed-pregnant SD rat embryos on the 17th day of gestation and mechanically dissociated into single cells by trituration with fire-polished Pasteur pipettes. The cerebrocortical cells containing neuronal and non-neuronal cells were seeded on either 35-mm culture dishes or 24-well plates, pre-coated with a mixture of laminin and poly-L-lysine. The respective seeding density was 6 × 10^6^ cells/dish or 6 × 10^5^ cells/well in MEM supplemented with 25 mM glucose, 2 mM glutamine, 5% HS, 5% FBS, and 1% antibiotic–antimycotic agent. The cells were maintained at 37 °C in an incubator with a humidified atmosphere of 95% air and 5% CO_2_. On the 7th day of seeding, the cells were treated with 10 μM cytosine arabinoside to arrest the proliferation of non-neuronal cells. The experiments were performed 10–11 days after plating.

### 2.5. Treatment of Cells with Various Types of Insults and Assessment of Cell Viability

Cultured cells were gently washed with N-(2-hydroxyethyl)piperazine-N-2-ethanesulfonic acid (HEPES)-buffered control salt solution (HCSS) (20 mM HEPES, pH 7.4; 5.4 mM KCl; 120 mM NaCl; 2.3 mM CaCl_2_·2H_2_O; 1.6 mM MgCl_2_·6H_2_O; 10 mM NaOH, and 15 mM glucose), and excitotoxic neurotoxicity was induced by the exposure to 100 μM glutamate in HCSS or 300 μM NMDA in Mg^2+^-free HCSS for 15 min [27,28]. The control cells were respectively treated with HCSS or Mg^2+^-free HCSS without any agent. The cells were then washed twice with HCSS and maintained in MEM supplemented with 25 mM glucose (MEMG) for 22–24 h in the incubator. To induce neurotoxicity by Aβ treatment, the cells were exposed to aggregated Aβ_(25–35)_, prepared as previously described [29], at a final concentration of 40 μM in MEMG for 24 h at 37 °C [28]. The control cells were treated with MEMG without any agent. To investigate the effects of MPW on the neurotoxicity induced by Glu, NMDA, or Aβ_(25–35)_, the cells were simultaneously treated with various concentrations of MPW in combination with the respective insults.

The stock solution of MPW was prepared in sterilized distilled water at the concentration of 30 mg/mL and appropriately diluted prior to the treatment. Following the desired treatments, cell viability was assessed using the MTT reduction assay, as previously described [26,28]. Briefly, MTT was added to the culture of the treated cells at a final concentration of 1 mg/mL in phosphate-buffered saline (PBS), and then the cells were incubated for 3 h at 37 °C. After removing the culture media, 500 µl dimethylsulfoxide (DMSO) was added to each well, and the formazan crystal products were dissolved by incubating at 37 °C for 15 min. The absorbance was measured at 550 nm by a microplate reader (SpectraMax M2^e^, Molecular Devices, Sunnyvale, CA, USA). The cell viability was expressed as a percentage of control-treated cells.

### 2.6. Measurement of Intracellular ROS Levels

Intracellular ROS levels in the absence or presence of MPW were spectrofluorometrically evaluated using the fluorogenic dye DCFH-DA as a probe [26]. Briefly, the cultured cells were washed with HCSS and then treated with DCFH-DA at a final concentration of 10 µM in MEMG for 30 min at 37 °C. Subsequently, the cells were washed with HCSS and then subjected to the corresponding insult (100 μM glutamate, 300 μM NMDA, or 40 μM Aβ_(25–35)_) in MEMG for 2 h in the absence or presence of various concentrations of MPW. Intracellular ROS levels were assessed by measuring the level of 2′,7′-dichlorofluorescein–derived fluorescence in a microplate reader (SpectraMax M2^e^, Molecular Devices) with the excitation and emission wavelengths at 490 nm and 520 nm, respectively. Intracellular ROS levels were expressed as a percentage of the level determined in the control-treated cells.

### 2.7. TUNEL Assay

The effect of MPW on the NMDA- or Aβ_(25–35)_-induced apoptosis was assessed by the detection of fragmented DNA using a TUNEL assay kit (DeadEnd™ Colorimetric TUNEL System, Promega, Madison, WI, USA), as previously described [26,30]. In brief, the cells were treated with 100 μM NMDA or 40 μM Aβ_(25–35)_ in MEMG for 2 h in the absence or presence of MPW (30 μg/mL), washed with PBS, and then fixed in 4% paraformaldehyde for 25 min at 4 °C. The cells were washed with PBS and then incubated in 0.2% Triton X-100 for 5 min for permeabilization. Following another round of washing, the cells were incubated in the equilibration buffer for 10 min at room temperature and then treated with the terminal deoxynucleotidyl transferase reaction mixture, consisting of the biotinylated nucleotide mix, for 60 min to permit the nick-end-labeling reaction. After the termination of the reaction, the cells were immersed in the saline sodium citrate solution and then incubated with streptavidin-conjugated HRP. The cells were washed twice with PBS and stained with diaminobenzidine. Finally, after washing the cells with PBS again, the TUNEL-positive cells stained in dark brown color were detected using a TS-100 inverted microscope (Nikon, Tokyo, Japan). The TUNEL-positive cells were manually counted from four randomly selected fields and expressed as percentages of total cell numbers, as described [26,30]. The counting was performed blind.

### 2.8. Western Blotting

Western blotting analysis was performed in the absence or presence of MPW as previously described [31]. Briefly, the cells cultured in 35 mm dishes were serum-starved overnight, treated with 100 μM NMDA for 12 h or with 40 μM Aβ_(25–35)_ for 18 h in MEMG with or without different concentrations of MPW. The cells were then lysed for 30 min on ice in the lysis buffer (150 mM NaCl; 10 mM Tris-HCl, pH 7.4; 2 mM EDTA; 10 mM β-glycerophosphate; 4.5 mM sodium pyrophosphate; 1 mM Na_3_VO_4_; 1 mM NaF; 0.5% (*v*/*v*) NP-40; 1% (*v*/*v*) Triton X-100; and one tablet of protease inhibitor cocktail (Roche Diagnostic GmbH, Mannheim, Germany)). The resultant lysates were centrifuged at 14,000 rpm for 30 min at 4 ℃, and then the supernatants were collected. After determining the total protein concentrations of the supernatants, equal amounts of lysate proteins (30 µg) were resolved on 12% gels via sodium dodecyl sulfate-polyacrylamide gel electrophoresis and electrophoretically transferred onto nitrocellulose membranes (Whatman, Clifton, NJ, USA) for 1.5 h at 100 V. Then, the membranes were blocked for 1.5 h with Tris-buffered saline (TBS) containing 0.1% Tween 20 (TBST) and 5% non-fat dry milk (BD Falcon, Sparks, MD, USA) and incubated overnight at 4 ℃ with anti-caspase 3 antibody in TBST containing 5% bovine serum albumin (USB, Canton, OH, USA). Subsequently, the membranes were washed three times with TBST and then incubated for 1.5 h with appropriate HRP-conjugated anti-rabbit IgG antibody. The immunoreactive bands were detected using Clarity^TM^ Western ECL substrate (Bio-Rad, Hercules, CA, USA) in the BioRad ChemiDoc XRS imaging system (BioRad).

### 2.9. Assessment of the LPO Levels in Rat Brain Homogenates

The effect of MPW on the LPO levels in rat forebrain homogenates was examined as previously described [32]. Briefly, an aliquot of SD rat forebrain homogenate and various concentrations of MPW were added to a reaction mixture containing 10 μM Fe^2+^ and 100 μM L-ascorbic acid and incubated at 37 °C for 1 h to allow the lipid peroxidation reaction to proceed. After stopping the reaction by adding 28% *w*/*v* trichloroacetic acid and 1% *w*/*v* TBA, the mixture was heated at 100 °C for 15 min and centrifuged at 3000 rpm for 10 min at 4 °C to remove the precipitates. The absorbance of the supernatant was measured at 532 nm on a microplate reader (SpectraMax M2^e^, Molecular Devices). The percent inhibition of the LPO was quantitated using the following equation:

Inhibition (%) = 100 × (1 − Abs_sample_/Abs_control_)

where Abs_control_ and Abs_sample_ denote the absorbance of the control (without MPW) and experimental sample (with MPW), respectively.

### 2.10. DPPH Radical Scavenging Activity Assay

DPPH radical scavenging activity in the absence or presence of MPW was measured as previously described [32]. Briefly, the reaction mixture containing various concentrations of MPW and methanolic solution of DPPH (150 µM) was incubated at 37 °C for 30 min. The absorbance was then measured at 520 nm on a microplate reader (SpectraMax M2^e^, Molecular Devices). The radical scavenging activity was determined using the following equation:

Radical scavenging activity (%) = 100 × (Abs_control_ − Abs_sample_)/Abs_control_

where Abs_control_ and Abs_sample_ denote the absorbance of the control (without MPW) and experimental sample (with MPW), respectively.

### 2.11. In Vitro Assessment of β-Secretase Activity

β-Secretase activity in the absence or presence of MPW was measured using a β-secretase FRET assay kit (Invitrogen, Carlsbad, CA, USA) according to the instructions of the manufacturer with some modifications [26]. In brief, 10 μL of the assay buffer containing various concentrations of MPW was added to 20 μL of the substrate (750 nM) into a 96-well plate. Then, 10 μL of β-secretase (1.0 U/mL) was added to the reaction mixture and incubated for 2 h at room temperature. The fluorescence was measured using a microplate reader (SpectraMax M2^e^, Molecular Devices) with the excitation and emission wavelengths at 545 and 585 nm, respectively.

### 2.12. In Vitro Assessment of Acetylcholinesterase (AChE) Activity

AChE activity in the absence or presence of MPW was measured according to the method of Ellman, with some modifications [33]. In brief, a reaction mixture consisting of 100 μL of 10 μM 5,5′-dithiobis (2-nitrobenzoic acid), 10 μL of 75 mM acetylthiocholine iodide, and 10 μL of various concentrations of MPW in phosphate buffer (pH 8.9) was incubated at 37 °C for 10 min. Subsequently, 100 μL of 0.2 U/mL AChE enzyme was added, and the mixture was incubated for 15 min at 37 °C. The absorbance of the mixture was read at 410 nm by a microplate reader (SpectraMax M2e, Molecular Devices). The percent inhibition of the AChE activity was quantitated using the following equation:

Inhibition (%) = 100 × (1 − Abs_sample_/Abs_control_)

where Abs_control_ and Abs_sample_ denote the absorbance of the control (without MPW) and the experimental sample (with MPW), respectively.

### 2.13. The Morris Water Maze (MWM) Test

The MWM test was performed using a circular pool (120 cm in diameter and 45 cm in height), according to the procedures previously described with some modifications [34]. The pool was filled with water containing black food dye to the depth of 30 cm and placed under dim light. The water temperature was maintained at 22–23 °C throughout the experiment. The pool was conceptually divided into four quadrants, to which four different shapes of visual cues were attached. A white platform (10 cm in diameter and 29 cm high) was subsequently placed in one of the quadrants and submerged 1 cm below the water surface.

On the first day of the experiment, the mice were acclimated to the pool for 120 s in the absence of the platform. During the next four consecutive days (days 1–4) of training trials, the mice were given two trial sessions of 60 s each per day with the platform in place. Once the mice reached the platform within 60 s, they were permitted to stay on it for 10 s. If the mice could not find the platform within 60 s, they were placed on it for 10 s. After each training trial, the animals were delivered to their cages and dried under an infrared lamp. The interval time between each trial session was 30 min. During each training trial session, the time to find the hidden platform (latency time) was recorded using a video camera-based EthoVision Maze Test System (Noldus Information Technology, Wageningen, Netherlands). On day 5 of the experiment (probe trial), the platform was removed, and the mice were allowed to swim for 60 s to search for it. The swimming time the mice spent in the target quadrant where the platform had previously been placed and the total swimming distance were recorded during the probe trial sessions.

To test the effects of MPW on memory impairment, mice were randomly divided into seven groups of 8 mice, and MPW prepared in normal saline was orally administered at the dosages of 10, 50, 100, and 300 mg/kg from day 1 to day 4, respectively. Donepezil was also administered at 10 mg/kg to serve as a positive control. After 30 min of MPW or donepezil administration, intraperitoneal injection of scopolamine (2 mg/kg) was used to induce memory impairment. After 30 min of scopolamine administration, training trials were performed each day from day 1 to day 4, as described above. On day 5, the probe trial was performed after the hidden platform was removed. The control group received vehicle (normal saline) only.

### 2.14. Statistical Analysis

All the experiments were carried out at least three times independently. Quantitative data are expressed as the mean ± S.E.M. Statistical analyses were performed using one-way ANOVA followed by Tukey’s post hoc test with SigmaPlot 12.5 software (Systat Software, San Jose, CA, USA). *p* < 0.05 was considered statistically significant.

## 3. Results

### 3.1. Preparation and Analysis of MPW

MPW was prepared from the dried pericarps of *Garcinia mangostana* L. and analyzed via UPLC-UV and Mass spectrometry, as described above. The yield of MPW was 3.8% (*w*/*w*). The spectroscopic chromatograms of MPW from UPLC-UV and Mass analyses exhibited several prominent peaks as well as many minor peaks (see Supplementary Materials). Among the peaks detected, both α- and γ-mangostins were found to be the major constituents of MPW. In addition, the peaks representing mangostanin and mangostanol were also observed along with several unidentified minor peaks. In this study, we evaluated the protective effects of MPW against the oxidative neurotoxicity triggered by various insults, such as Aβ or excitotoxic amino acids, in primary rat cortical cells. Furthermore, we investigated the memory-improving effect of MPW on scopolamine-induced impairment of spatial learning and memory in mice.

### 3.2. Effects of MPW on Aβ_(25__–35)_-Induced Neurotoxicity and ROS Generation in Primary Rat Cortical Cells

**Figure 1:**
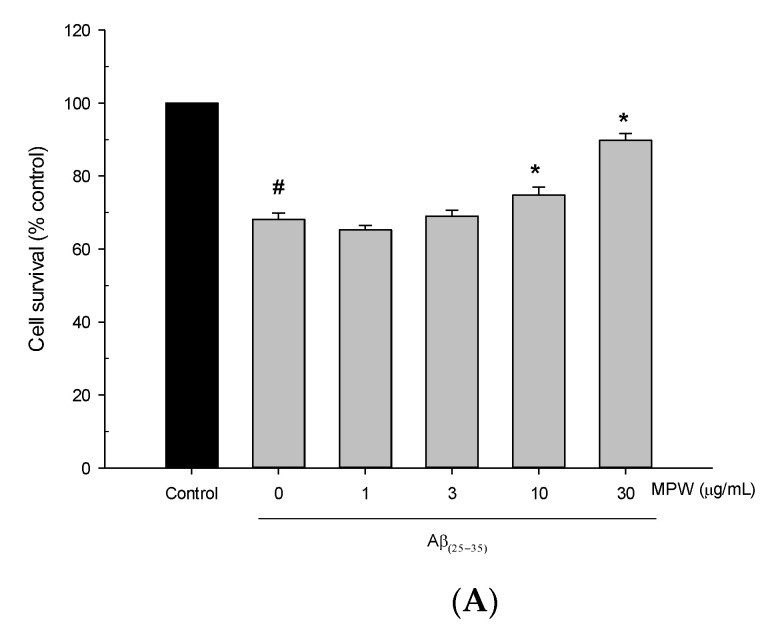

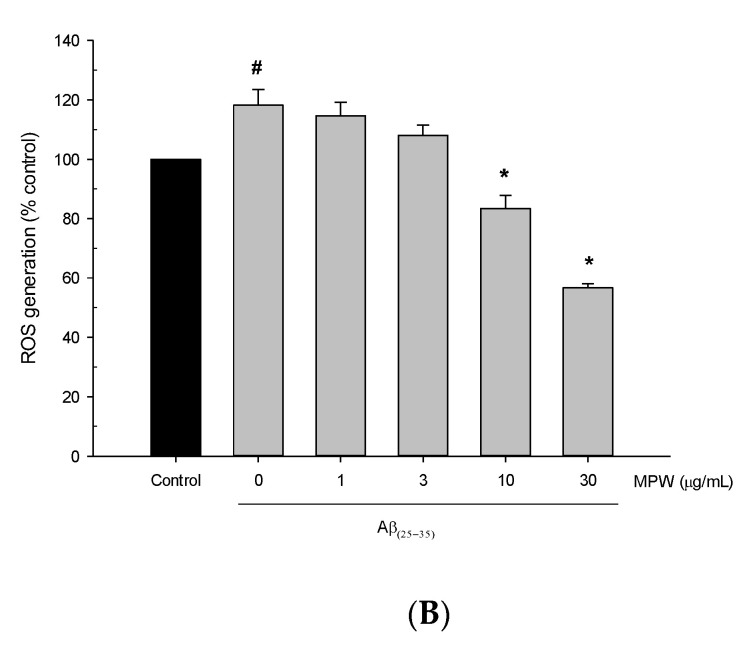
Effects of MPW on Aβ_(25__–35)_-induced neurotoxicity and ROS generation in primary rat cortical cells. (**A**) The cultured cells were exposed to 40 μM Aβ_(25__–35)_ in MEMG for 24 h in the absence or presence of MPW at the indicated concentrations. The cell viability was determined by the MTT reduction assay at 22–24 h of the exposure, as described in the Materials and Methods. The viability of each treated group is expressed as a percentage of that of the vehicle-treated control cells. (**B**) The cultured cells were pre-incubated with 10 μM DCFH-DA for 30 min at 37 ℃ in the dark and then treated with 40 μM Aβ_(25__–35)_ in MEMG in the absence or presence of MPW at the indicated concentrations. The intracellular ROS levels were measured by the fluorescence detection of 2′,7′-dichlorofluorescein, as described in the Materials and Methods. The ROS levels in each treated group are expressed as percentages of those in the vehicle-treated control cells. Each data point represents the mean ± S.E.M. from at least three independent experiments conducted in duplicate (#, *p* < 0.05 vs. the vehicle-treated control cells without MPW; *, *p* < 0.05 vs. the Aβ_(25__–35)_-treated cells without MPW).

To examine the neuroprotective action by MPW, we first tested its effect on the neurotoxicity induced by Aβ_(25__–35)_, the active fragment of Aβ, in primary rat cortical cells. Treatment of the cells for 24 h with 40 μM Aβ_(25__–35)_ decreased the cell viability by approximately 30%, compared to the viability of the vehicle-treated control cells (Figure 1A). The reduced viability of the Aβ_(25__–35)_-treated cells was significantly reversed by MPW at the concentrations of 10 μg/mL and above (Figure 1A). In addition, treatment of the cells with Aβ_(25__–35)_ for 2 h increased intracellular ROS production, which was markedly attenuated by MPW (Figure 1B).

### 3.3. Effects of MPW on Aβ_(25__–35)_-Induced Caspase 3 Activation and DNA Fragmentation in Primary Rat Cortical Cells

**Figure 2:**
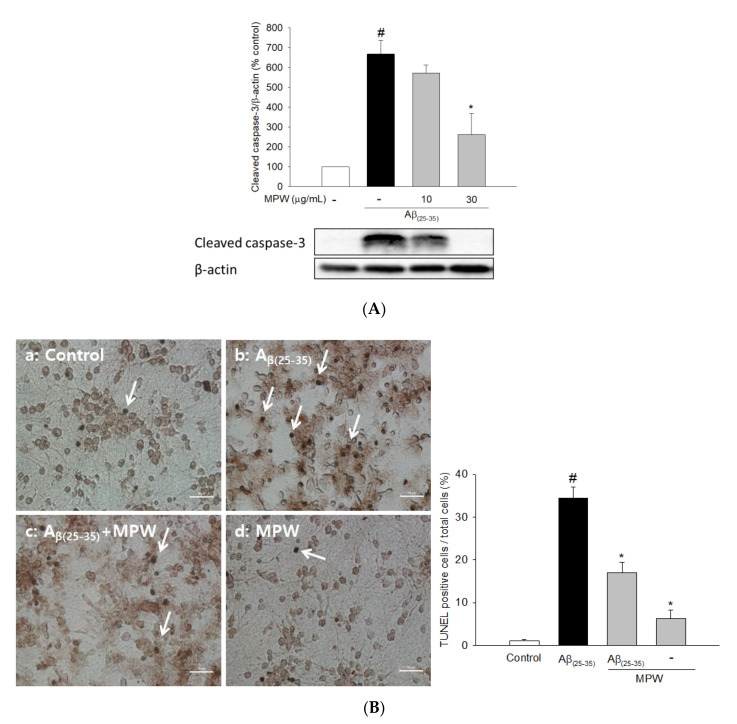
Effects of MPW on Aβ_(25__–35)_-induced caspase 3 activation and DNA fragmentation in primary rat cortical cells. (**A**) The cultured cells were treated with 40 μM Aβ_(25__–35)_ for 24 h in the absence or presence of MPW at 10 or 30 μg/mL. The level of cleaved caspase 3 was assessed by Western blotting, as described in the Materials and Methods. A representative blot from three independent experiments is shown. (**B**) The cultured cells were treated with 40 μM Aβ_(25__–35)_ for 2 h in the absence or presence of MPW at 30 μg/mL, and the TUNEL assay was carried out as described in the Materials and Methods. Representative microscopic images from at least three independent experiments are shown. Cells were treated with the vehicle (a), Aβ_(25__–35)_ (b), Aβ_(25__–35)_ and MPW (c), or MPW alone (d), respectively. The representative TUNEL-positive cells are indicated by white arrows. Scale bar = 10 μm. Each data point represents the mean ± S.E.M. from at least three independent experiments (#, *p* < 0.05 vs. the vehicle-treated control cells without MPW; *, *p* < 0.05 vs. the Aβ_(25__–35)_-treated cells without MPW).

To elucidate the probable mechanism(s) by which MPW suppressed A_β__(25–35)_-induced neurotoxicity, we examined the effect of MPW on the Aβ_(25__–35)_-induced apoptosis in primary rat cortical cells. Consistent with the previous study [26], the exposure of cultured cells to Aβ_(25__–35)_ dramatically increased the level of cleaved caspase 3, one of the major intracellular signals representing apoptotic processes (Figure 2A). The A_β__(25–35)_-induced caspase 3 activation was markedly decreased by MPW. In addition, treatment of the cultured cells with A_β__(25–35)_ significantly increased DNA fragmentation, another important hallmark of apoptotic processes. Again, the increased TUNEL-positive cell population with DNA fragmentation was remarkably reduced by MPW at 30 μg/mL (Figure 2B).

### 3.4. Effects of MPW on Glutamate- or NMDA-Induced Neurotoxicity and ROS Generation in Primary Rat Cortical Cells

**Figure 3:**
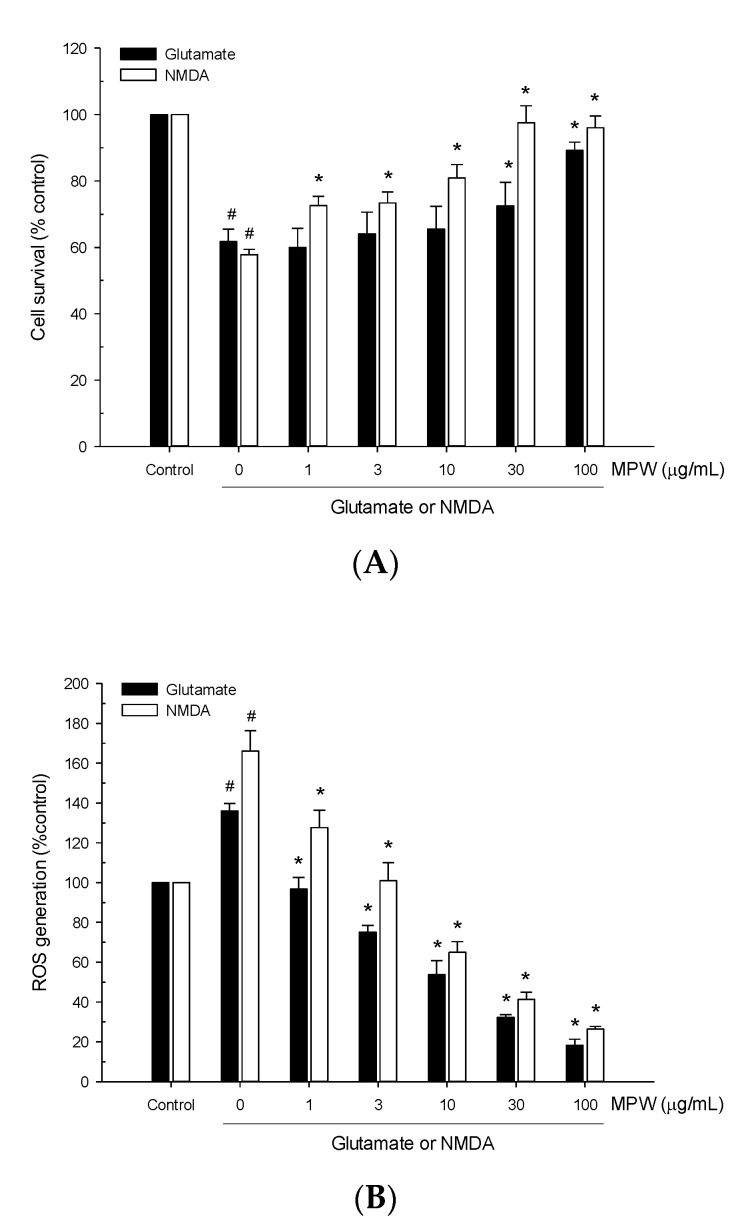
Effects of MPW on glutamate- or NMDA-induced neurotoxicity and ROS generation in primary rat cortical cells. (**A**) The cultured cells were exposed to 100 μM glutamate or 300 μM NMDA for 15 min in the absence or presence of MPW at the indicated concentrations. The cell viability was determined by the MTT reduction assays, and the cell survival was calculated as described in Figure 1. (**B**) The cultured cells were pre-incubated with 10 μM DCFH-DA for 30 min at 37 ℃ in the dark and then treated with 100 μM glutamate or 300 μM NMDA for 2 h in the absence or presence of MPW at the indicated concentrations. The intracellular ROS levels were measured by the fluorescence detection of 2′,7′-dichlorofluorescein and calculated as described in Figure 1. Each data point represents the mean ± S.E.M. from at least three independent experiments conducted in duplicate (#, *p* < 0.05 vs. the vehicle-treated control cells without MPW; *, *p* < 0.05 vs. the glutamate- or NMDA-treated cells without MPW).

We next evaluated the effect of MPW on excitotoxic neurotoxicity induced by glutamate or NMDA in primary rat cortical cells. The cells were treated with 100 μM glutamate or 300 μM NMDA for 15 min in the absence or presence of MPW at various concentrations, and the cell viability was assessed by the MTT reduction assay at 22–24 h of the exposure. As illustrated in Figure 3A, treatment of the cells with glutamate or NMDA reduced the cell viability by approximately 40%, compared to the viability of the vehicle-treated control cells. The reduced viability of the glutamate- or NMDA-treated cells was reversed by MPW treatment (Figure 3A). While the glutamate-induced toxicity was significantly suppressed by MPW at the concentrations of 30 μg/mL and above, the NMDA toxicity was significantly suppressed by MPW at as low as 1 μg/mL and completely reversed at 30 μg/mL and above.

Additionally, treatment of cells with glutamate or NMDA increased the intracellular ROS levels to approximately 140% or 170%, respectively, compared to the vehicle-treated control cells (Figure 3B). The glutamate- or NMDA-induced increases in ROS production were inhibited by MPW in concentration-dependent manners (Figure 3B).

### 3.5. Effects of MPW on NMDA-Induced Caspase 3 Activation and DNA Fragmentation in Primary Rat Cortical Cells

**Figure 4:**
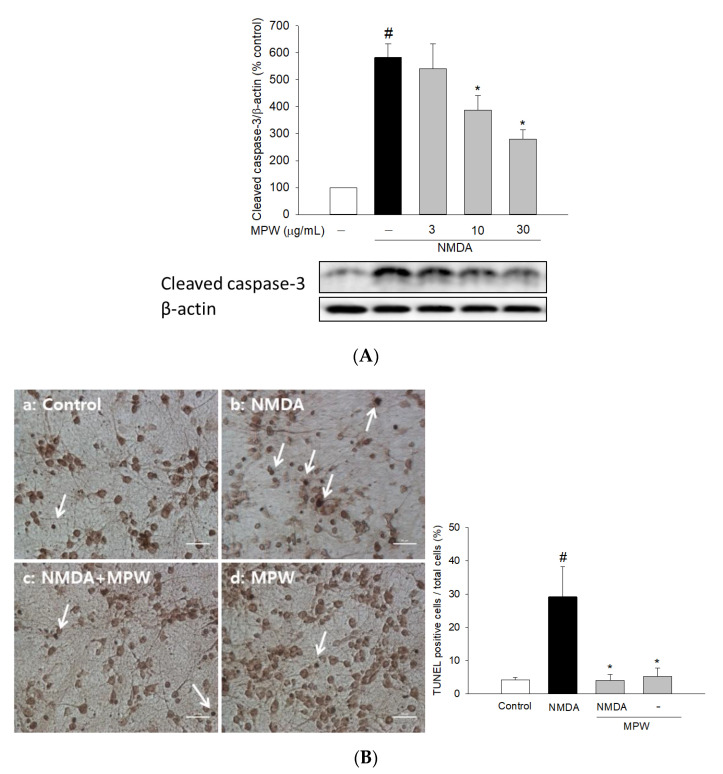
Effects of MPW on NMDA-induced caspase 3 activation and DNA fragmentation in primary rat cortical cells. (**A**) The cultured cells were treated with 100 μM NMDA for 12 h in the absence or presence of MPW at 3, 10, and 30 μg/mL. The level of cleaved caspase 3 was assessed by Western blotting, as described in the Materials and Methods. A representative blot from three independent experiments is shown. (**B**) The cultured cells were treated with 100 μM NMDA for 2 h in the absence or presence of MPW at 30 μg/mL, and then the TUNEL assay was carried out. Representative microscopic images from at least three independent experiments are shown. Cells were treated with the vehicle (a), NMDA (b), NMDA and MPW (c), or MPW alone (d), respectively. The representative TUNEL-positive cells are indicated by white arrows. Scale bar = 10 μm. Each data point represents the mean ± S.E.M. from at least three independent experiments (#, *p* < 0.05 vs. the vehicle-treated control cells without MPW; *, *p* < 0.05 vs. the NMDA-treated cells without MPW).

We then examined the effect of MPW on the NMDA-induced apoptosis. In agreement with the previous report [26], the exposure of cultured cells to NMDA markedly increased the level of cleaved caspase 3, and this effect was suppressed by MPW treatment (Figure 4A). Similarly, DNA fragmentation triggered by NMDA was remarkably reduced by MPW at 30 μg/mL (Figure 4B).

Collectively, our findings indicated that MPW exhibited neuroprotective action against Aβ_(25__–35)_-induced neurotoxicity as well as glutamate- or NMDA-induced excitotoxicity through the inhibition of both ROS generation and apoptosis.

### 3.6. Effects of MPW on LPO and DPPH Radical Formation

**Figure 5:**
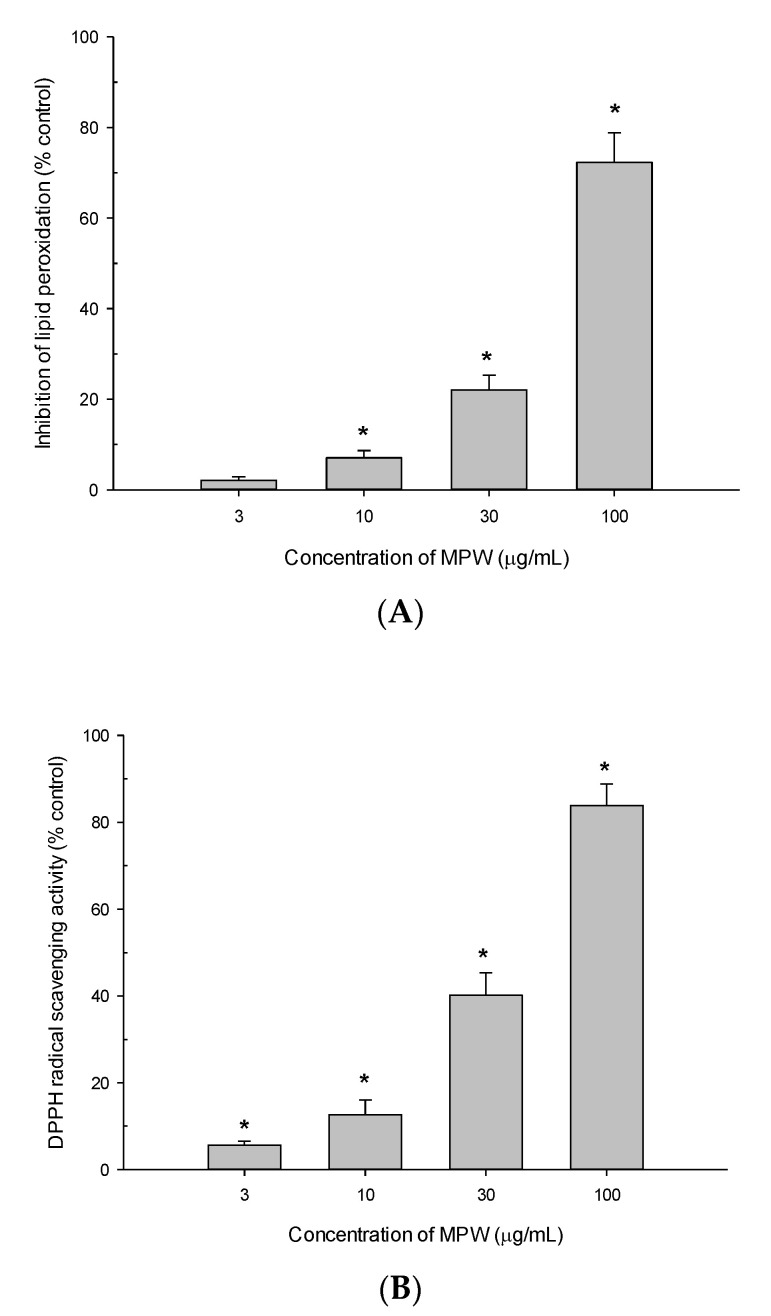
Effects of MPW on LPO and DPPH radical formation. Inhibition of LPO induced by Fe^2+^ (10 µM) and L-ascorbic acid (100 µM) in rat forebrain homogenates (**A**) and DPPH radical formation (**B**) by MPW at the indicated concentrations were measured as described in the Materials and Methods. Each data point represents the mean ± S.E.M. from at least three independent experiments conducted in duplicate (*, *p* < 0.05 vs. LPO or DPPH radicals measured in the absence of MPW).

The antioxidant properties of MPW were further investigated by evaluating its ability to inhibit LPO initiated by Fe^2+^ and L-ascorbic acid in rat brain homogenates. In addition, the radical scavenging activity of MPW was assessed using stable free radical DPPH as a probe. Our results demonstrated that MPW considerably inhibited lipid peroxide formation in rat brain homogenates (Figure 5A) and effectively scavenged DPPH radicals (Figure 5B) in concentration-dependent manners. The calculated IC_50_ values were 58.9 and 37.1 μg/mL, respectively.

### 3.7. Effect of MPW on β-Secretase Activity

**Figure 6:**
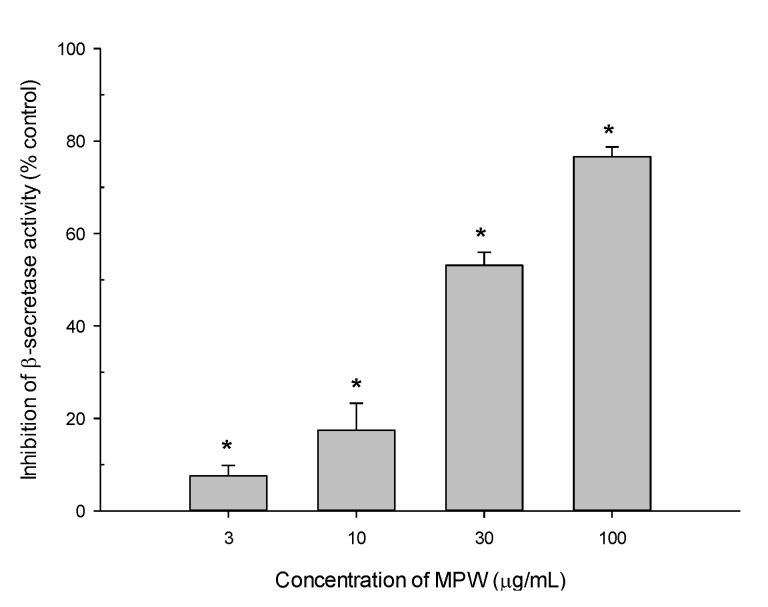
Effect of MPW on β-secretase activity. The β-secretase enzyme activities were determined using the in vitro β-secretase FRET assay in the absence or presence of MPW at various concentrations, as described in the Materials and Methods. The data are expressed as percentages of inhibition of the enzyme activity measured in the absence of MPW. Each data point represents the mean ± S.E.M. from at least three independent experiments conducted in duplicate (*, *p* < 0.05 vs. the enzyme activity measured in the absence of MPW).

Aβ peptide is derived from APP upon enzymatic cleavage by β- and γ-secretases [1,2]. To evaluate the impact of MPW on the synthesis of Aβ peptide, we measured β-secretase activity in the presence of various concentrations of MPW using an in vitro β-secretase FRET assay kit. We found that MPW inhibited β-secretase activity, with an IC_50_ value of 31.0 μg/mL (Figure 6).

### 3.8. Effect of MPW on AChE Activity

**Figure 7:**
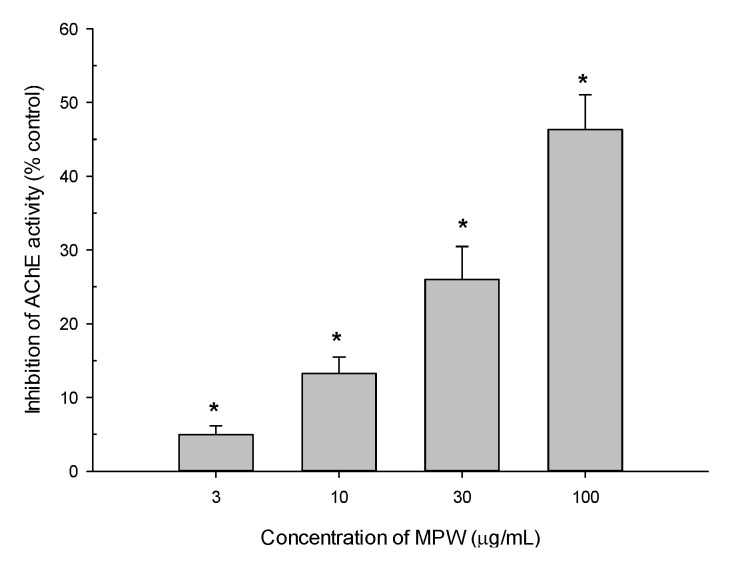
Effect of MPW on AChE activity. The AChE enzyme activities were determined using Ellman’s method with some modifications in the absence or presence of MPW at various concentrations, as described in the Materials and Methods. The data are expressed as percentages of inhibition of the enzyme activity measured in the absence of MPW. Each data point represents the mean ± S.E.M. from at least three independent experiments conducted in duplicate (*, *p* < 0.05 vs. the enzyme activity measured in the absence of MPW).

We also evaluated the effect of MPW on AChE activity, determined using Ellman’s colorimetric method [33]. As shown in Figure 7, MPW significantly and concentration-dependently inhibited AChE activity at the concentrations tested. The maximal inhibition achieved at 100 μg/mL of MPW was approximately 50% of the enzyme activity measured in the absence of MPW.

### 3.9. Effect of MPW on Scopolamine-Induced Spatial Learning and Memory Impairment in Mice

**Figure 8:**
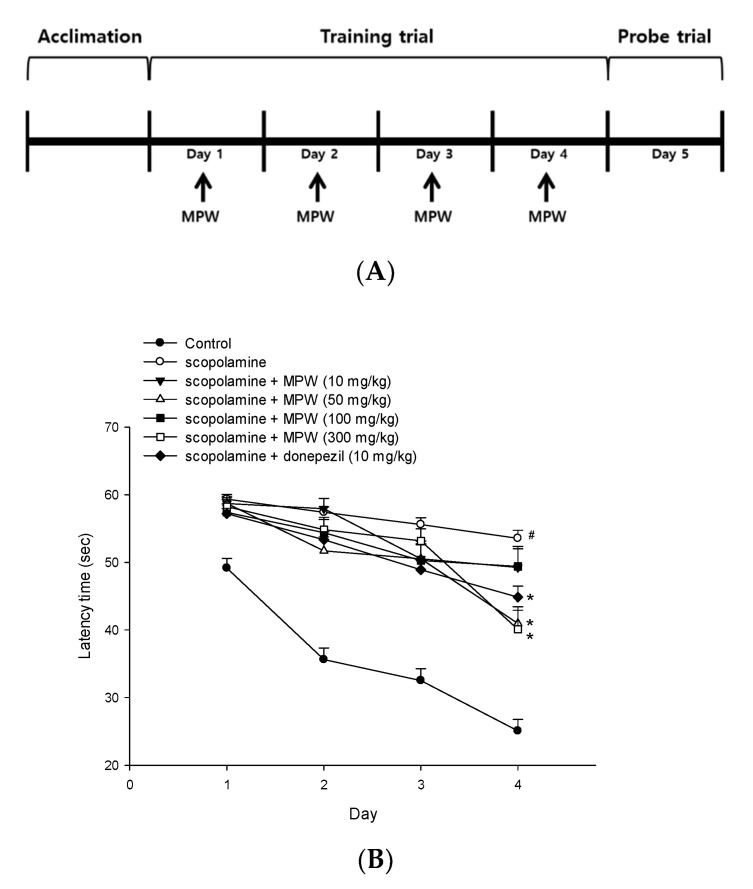

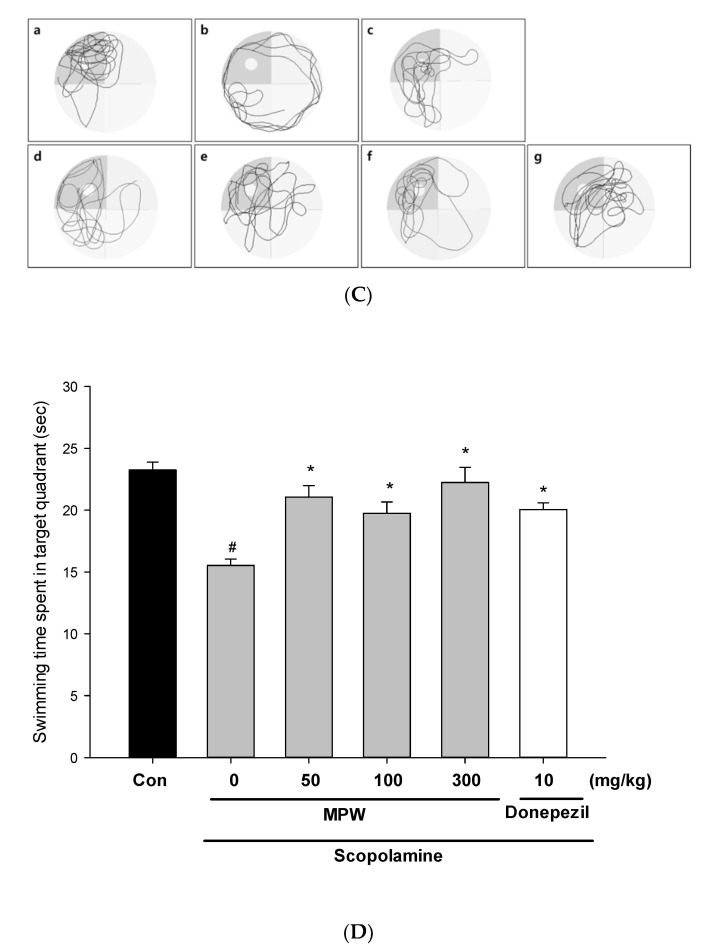
Effect of MPW on scopolamine-induced spatial learning and memory impairment in mice. Animals were randomly divided into 7 groups (8 mice per group). MPW was orally administered at the dosages of 10, 50, 100, and 300 mg/kg from day 1 to day 4, respectively, and training and probe trials were performed. For the reference group, donepezil was administered at the dosage of 10 mg/kg. After 30 min of each administration, intraperitoneal injection of scopolamine (2 mg/kg) was used to induce spatial learning and memory impairment. After 30 min of scopolamine injection, the water maze test was performed as described in the Materials and Methods. (**A**) A schematic diagram indicating the timeline of MPW administration and training and probe trials is shown. (**B**) During the training trials performed on days 1–4, the latency time, the time to find the hidden platform, was determined for each group. (**C**,**D**) On the following day (day 5), the probe trial was performed after the hidden platform was removed. The representative images tracking the swimming paths of control (a), scopolamine (b), scopolamine + MPW 10 mg/kg (c), scopolamine + MPW 50 mg/kg (d), scopolamine + MPW 100 mg/kg (e), scopolamine + MPW 300 mg/kg (f), and scopolamine + donepezil 10 mg/kg (g) group, respectively, are shown (**C**). During the probe trial, the swimming time in the target quadrant where the platform had previously been located was determined (**D**). The control group received vehicle (normal saline) only. Each data point represents the mean ± S.E.M. (#, *p* < 0.05 vs. the vehicle-treated control group; *, *p* < 0.05 vs. the scopolamine-injected group).

Based on our findings from cell-based and cell-free in vitro assays in this study, we finally carried out the MWM test to examine whether MPW could improve spatial learning and memory impairment induced by scopolamine in mice. MPW was orally administered once daily at the dosages of 10, 50, 100, and 300 mg/kg for 4 consecutive days, as illustrated in Figure 8A. The latency time to reach the platform was measured on each day of the training trial at 1 h after MPW administration (Figure 8B). The latency time in the vehicle-treated control group showed a dramatic decrease from day 1 to day 4. In contrast, the latency time in the scopolamine group was not considerably changed during 4 days of the training trial, indicating that spatial learning capability of mice was impaired by scopolamine injection. The latency time in groups administered with MPW or donepezil, a positive reference drug, showed a tendency to be decreased during the training trial, as compared to that of the scopolamine group. Particularly, the MPW groups (50 and 300 mg/kg) and the donepezil group (10 mg/kg) significantly shortened the latency time on day 4 (*, *p* < 0.05 vs. the scopolamine group; Figure 8B).

On the following day (day 5), the probe trial was performed after removing the platform. The representative images tracking the swimming paths are provided (Figure 8C). During the probe trial, the swimming time each mouse spent in the target quadrant where the platform had previously been located is shown (Figure 8D). The swimming time the scopolamine-treated mice spent in the target quadrant was markedly decreased relative to that of the control group (#, *p* < 0.05 vs. the control group), indicating that spatial memory function was also impaired in this group. However, the time spent in the target quadrant was significantly restored in the groups treated with MPW at the dosages of 50 mg/kg and above. The reduced swimming time of the scopolamine group was also recovered by donepezil administration. Interestingly, MPW administration (50–300 mg/kg of dosages) recovered scopolamine-induced spatial memory impairment to the same extent as, or even better than, that of the donepezil group. MPW at 10 mg/kg did not show a significant effect (Figure 8D). The total swimming distances measured on day 5 in all the test groups were not significantly affected by MPW or donepezil treatment (data not shown).

## 4. Discussion

Mangosteen has been utilized as a traditional medicine to treat various health problems, such as wounds, infections, inflammation, and diarrhea, in Southeast Asia [35]. Mangosteen pericarp and its bioactive xanthones have been documented to elicit biological actions in the brain, and thus, may exhibit beneficial effects to prevent or treat various brain disorders, including AD, Parkinson’s disease, and depression [36]. Diverse extracts, such as methanol, chloroform, and 50% ethanol extracts, prepared from mangosteen pericarp were reported to exhibit a wide variety of biological activities, including antioxidant, anti-inflammatory, analgesic, anti-cancer, and neuroprotective effects [15,20]. However, the antioxidant and neuroprotective effects of the water extract (MPW) have not been reported yet. Since MPW has been prepared without using any organic solvents, it is expected to have advantages with regard to safety concerns when administered to animals and humans. In this study, we prepared MPW from dried mangosteen pericarps and used primary cultured rat cortical cells to investigate its effects on the neurotoxicity triggered by various insults. Furthermore, we also evaluated the effect of MPW on scopolamine-induced memory dysfunction in mice by the MWM test.

We revealed the protective effects of MPW against the neurotoxicity, oxidative stress, and apoptotic processes induced by numerous insults, such as Aβ_(__25–35__)_, glutamate, and NMDA. In addition, MPW possessed antioxidant properties, inhibiting LPO and scavenging DPPH radicals. Moreover, MPW was demonstrated to inhibit β-secretase and AChE activities, which are associated with the typical pathological features of AD. Finally, this study showed that the impairment of the spatial learning and memory function in the scopolamine-treated mice was markedly alleviated by orally administered MPW. Our findings suggest that MPW may exert memory-enhancing effects, presumably through antioxidative neuroprotection as well as anti-apoptotic action.

The formation and deposition of Aβ plaques are well-known to be one of the most commonly accepted hallmarks in the pathology of AD. Aβ peptides, after forming soluble oligomers, aggregate into insoluble beta-sheet conformations and are then extracellularly deposited into diffuse senile plaques. This process affects the neuron-to-neuron communications at the synapses, eventually resulting in neuronal cell death [36,37]. Notably, the synthetic peptide Aβ_(25–35)_, which corresponds to amino acids 25–35 in the Aβ_(1–40)_ and Aβ_(1–42)_ [38,39], retains most physical and biological properties of the full-length Aβ [40]. Particularly, with its rapid aggregation, Aβ_(25–35)_ is commonly used in many in vitro studies to investigate the neuroprotective effects of various drugs in modulating the toxicity of Aβ [41]. In this study, we found that the treatment of primary rat cortical cells with Aβ_(25__–35)_ dramatically decreased the cell viability (Figure 1A). The treatment with MPW suppressed the Aβ_(25__–35)_-induced cell death in a concentration-dependent manner (Figure 1A), suggesting the neuroprotective effect of MPW.

It has been reported that the neuronal cell damage induced by Aβ peptide is through the generation of oxidative stress [5]. Aβ oligomers may serve as a source of ROS and initiate LPO in brain cell membranes, which is toxic to neurons [42]. Consistently with these previous findings [5], we observed that the treatment of the cultured cortical cells with Aβ_(25–35)_ for 2 h significantly increased ROS production. The increase in ROS levels was dramatically inhibited by MPW (Figure 1B), indicating the antioxidative effect of MPW. A growing body of evidence has demonstrated that oxidative stress can induce mitochondrial dysfunction, which eventually triggers apoptosis. Increased ROS production promotes mitochondrial dysfunction, thereby leading to cytochrome C release [43]. Cytochrome C interacts with the factors Apaf-1 and caspase 9 and subsequently activates apoptosis-associated caspases. Especially, the activation of caspase 3 causes DNA fragmentation and apoptosis [44,45]. In this study, treatment of cultured cells with Aβ_(25__–35)_ dramatically enhanced apoptotic processes, with increased cleaved caspase 3 level and DNA fragmentation. The enhancement of these apoptotic signals was significantly reversed by MPW (Figure 2), indicating the anti-apoptotic action of MPW. Collectively, these results imply that MPW protects cortical neurons from the Aβ_(25–35)_-induced neurotoxicity and apoptosis presumably through antioxidative activities.

Glutamate is the crucial excitatory neurotransmitter in the CNS. The interaction between glutamate and its excitatory amino acid receptors is necessary for normal synaptic function. However, for any reason, if the receptor activation becomes prolonged or excessive, the target neurons will be damaged and ultimately die [9]. The excitotoxicity of glutamate is mediated, in most cases, by the interaction with NMDA receptors, which leads to uncontrollable rises of intracellular Ca^2+^ concentrations, resulting in cell lysis and death [46]. In the present study, the treatment of the cultured cells with glutamate or NMDA considerably suppressed the cell survival, compared to that of the vehicle-treated control cells (Figure 3A). The decreased viability of the glutamate- or NMDA-treated cells was reversed by MPW, suggesting the neuroprotective properties of MPW against excitotoxicity.

Excessive NMDA receptor activity also triggers a cascade of apoptotic-like cell death. NMDA receptor hyperactivation activates the p38 mitogen-activated kinase and then myocyte enhancer factor 2C (MEF2C). MEF2C is a transcription factor that influences neuronal injury and apoptosis. Additionally, overactivation of NMDA receptors accentuates the toxic effects of free radicals, including ROS and nitric oxide, and activates caspases and apoptosis-inducing factors [47]. Further evidence has demonstrated that the activation of NMDA receptors is required for Aβ-induced over-production of ROS [48], which eventually causes the neurodegeneration and memory dysfunction seen in the brains of AD patients [36,49,50]. Therefore, dysregulation of NMDA receptor activity and oxidative stress may play dual deleterious roles in AD [51]. Notably, our results showed that glutamate or NMDA treatment of the cells caused a significant increase in ROS production, which was considerably attenuated by MPW (Figure 3B). Moreover, it was revealed that MPW treatment reduced the apoptotic processes, including NMDA-induced caspase-3 activation and DNA fragmentation (Figure 4). These results indicate the protective effects of MPW against the neurotoxicity, oxidative stress, and apoptotic processes in glutamate- or NMDA-treated cells.

The antioxidant properties of MPW were further illustrated in cell-free bioassays, with the inhibition of LPO induced by Fe^2+^ and L-ascorbic acid in rat brain homogenates (Figure 5A) and scavenging activity of DPPH radicals (Figure 5B). These antioxidant effects of MPW may be correlated with its inhibitory effects on the ROS generation triggered by Aβ_(25__–35)_, glutamate or NMDA (Figure 1B and Figure 3B). Our findings are consistent with previous reports involving other extracts prepared from mangosteen pericarps. The 50% ethanol extract exhibited potent antioxidant effects with high free-radical scavenging and neuroprotective actions in H_2_O_2_-treated NG108-15 cells [23]. Interestingly, the water-soluble partition of methanol or ethanol extracts successfully protected SK-N-SH neuroblastoma cells from cytotoxicity and inhibited the increases in ROS levels and caspase 3 activity induced by Aβ_(1–42__)_ or H_2_O_2_ and polychlorinated biphenyls treatments, respectively [52]. Moreover, the antioxidant and anti-apoptotic activities of the water-soluble partition of the ethanol extract were observed in the brains of scopolamine-treated mice [52]. It would be interesting if we could compare the in vitro and in vivo efficacies of these extracts with those of MPW under the same experimental conditions and identify bioactive components in these preparations.

Spectroscopic chromatograms of MPW revealed that it contained α- and γ-mangostins and considerable amounts of mangostanin and mangostanol, along with several unidentified minor peaks (Supplementary Materials). The α- and γ-mangostins were shown to have neuroprotective activities against glutamate-induced HT22 hippocampal neuronal cell death by upregulating HO-1 protein level [53]. In another study, however, only γ-mangostin, not α-mangostin, was reported to protect the primary rat cortical cells against both H_2_O_2_- and xanthine/xanthine oxidase-induced oxidative neuronal death as well as ROS production [24]. This xanthone also showed its significant anti-apoptotic activities, suppressing H_2_O_2_-induced DNA fragmentation and activations of caspases-3 and -9. In contrast to α-mangostin, γ-mangostin effectively reduced DPPH radical formation and LPO in rat brain homogenates [24]. Although data related to the effects of mangostanin and mangostanol on neuronal cells are still limited, mangostanin was suggested to exhibit antioxidant effects through induction of quinone reductases in the Hepa 1c1c7 mouse hepatoma cells [54]. Therefore, it can be speculated that γ-mangostin and possibly α-mangostin and mangostanin may contribute, at least in part, to the antioxidative neuroprotection and memory-enhancing effects of MPW. However, further investigation is needed to identify the bioactive constituent(s) of MPW. Collectively, the MPW-induced antioxidative neuroprotection observed in our study is consistent with the effects of various mangosteen pericarp extracts and bioactive xanthones.

β-Secretase, along with γ-secretase, produces Aβ by sequentially cleaving APP. Inhibition of β-secretase is considered to be a potential strategy to lower cerebral Aβ levels to treat and prevent AD [55]. In this study, MPW potently inhibited β-secretase activity with an IC_50_ value of 31.0 μg/mL (Figure 6), suggesting a promising MPW-mediated suppression of Aβ production. Intriguingly, β-secretase activity was also abolished by xanthones from mangosteen pericarp, including α- and γ-mangostins [24,53]. Particularly, α-mangostin diminished Aβ_(1–40__)_ and Aβ_(1–42__)_ production through the inhibitions of β-secretase and possibly γ-secretase activities in the amyloidogenic pathway in primary rat cortical cells [56]. The inhibition of β-secretase activity by MPW observed in this study may also be due to its constituents including α- and γ-mangostins.

Moreover, the intellectual deficiency in AD patients has been linked to a selective loss of cholinergic function in specific parts of the brain, such as the cortex and hippocampus [57]. Therefore, the primary therapeutic strategy in the treatment of AD is based on the restoration of the cholinergic function [58]. The inhibitors of cholinesterase, especially AChE, are designed to attenuate the breakdown of acetylcholine and retain its activity at cholinergic synapses. Clinical applications of the currently approved cholinesterase inhibitors are generally considered symptomatic treatments to improve cognitive, daily, and global functions, and some behavioral manifestations of AD [59]. As shown in Figure 7, MPW significantly attenuated AChE activity at the concentrations tested in this study. From our preliminary experiments, we observed some, but significant toxicity in the cells treated with MPW for 24 h at the concentration of 300 μg/mL, while no toxicity was detected up to 100 μg/mL of MPW (data not shown). Accordingly, the effects of MPW were examined at the concentrations up to 100 μg/mL in this study. The inhibition of AChE activity by MPW at 100 μg/mL was approximately 50% of the enzyme activity measured in the absence of MPW (Figure 7). Growing evidence has demonstrated the inhibition of AChE activities by MPE or MPE components. The water-soluble partition of ethanol MPE dramatically diminished AChE activity of SK-N-SH cells [60]. Moreover, pretreatment with 50% ethanol MPE in streptozotocin-injected male SA mice induced a dose-dependent decrease in AChE activity in the brain [61]. Additionally, α-mangostin and mangostanol were reported to exhibit selective inhibition of AChE activity assessed by Ellman’s colorimetric method [62]. Since both α-mangostin and mangostanol have been found in MPW (Supplemental Materials), these components may be responsible for the inhibition of AChE activity. Taken together, our study demonstrated using various cell-based and cell-free in vitro models that MPW exhibited antioxidative and anti-apoptotic neuroprotection as well as inhibition of β-secretase and AChE activities.

Injection of scopolamine, an anti-muscarinic agent, is known to cause progressive impairment of learning and memory in animals [63,64]. Moreover, the scopolamine-induced memory deficit is associated with the enhancement of oxidative stress and induction of apoptotic processes in the brain [65,66]. Thus, scopolamine-induced amnesia is widely used as an animal model of memory dysfunction to evaluate potential effects of drugs for AD [66,67]. In this study, we also employed this scopolamine-induced amnesia model to evaluate the effect of orally administered MPW by using the MWM test (Figure 8A). We found that the administration of MPW or donepezil, a well-known AChE inhibitor clinically used to treat AD patients, significantly decreased the latency time during the training trial and markedly increased the time spent in the target quadrant (Figure 8B–D). Interestingly, the memory-enhancing effect of MPW at the dosages of 50–300 mg/kg was nearly comparable to or even better than that of the donepezil-treated group. Notably, similar results were previously reported for the water-soluble partition of ethanol extracts, which was also revealed to improve scopolamine-induced memory dysfunction in mice, as assessed via the MWM and passive avoidance tests [60]. Moreover, pretreatment of streptozotocin-injected mice with 50% ethanol extract increased the habituation memory and cognitive function, as measured by the open field and Y-maze tests [61]. Furthermore, mangosteen pericarp diet exhibited neuroprotective and antioxidant activities in triple transgenic AD mice. Hippocampal Aβ deposition was also attenuated, which might further diminish the impairment of spatial memory retrieval in the MWM test [68]. Considerably, the oral administration of γ-mangostin successfully reversed the scopolamine-induced memory deficits in mice [24]. Taken together, our in vitro and in vivo findings in the current study imply that MPW exerts memory-enhancing effects in scopolamine-treated mice, probably through its antioxidative neuroprotection and anti-apoptotic action.

## 5. Conclusions

In this study, we used primary cultured rat cortical cells to investigate the effect of MPW on neurotoxicity induced by various insults. We found that MPW inhibited neurotoxicity and production of intracellular ROS triggered by Aβ_(25–35)_ or excitatory amino acids. MPW inhibited caspase 3 activation and DNA fragmentation in Aβ_(25–35)_- or NMDA-treated cells, suggesting that MPW has an anti-apoptotic action. Additionally, MPW inhibited LPO and scavenged DPPH radicals, thus assuring its antioxidant properties. Moreover, MPW suppressed β-secretase and AChE activities, which might also influence its effect on memory function. Finally, the oral administration of MPW significantly reversed the memory impairment in scopolamine-treated mice, with decreased latency time to find the platform and markedly increased swimming time in the target quadrant. These findings demonstrated the antioxidative neuroprotection and anti-apoptotic action of MPW, and thereby the spatial memory function of scopolamine-treated mice was improved. Accordingly, our study provides in vitro and in vivo preclinical evidence to support therapeutic potential of mangosteen pericarp, particularly its water extract, for prevention or treatment of cognitive impairment associated with AD.

## Supplementary Materials

The following are available online at https://www.mdpi.com/2076-3921/10/1/34/s1, Figure S1: UPLC-UV chromatogram of mangosteen water extract at 254 nm wavelength, Figure S2: UPLC-UV chromatogram of mangosteen water extract at 270 nm wavelength, Figure S3: Mass chromatogram of mangosteen water extract, Figure S4: Mass spectrometric data of individual peaks at different retention time.
