# Supplementary material for: Memory-Enhancing Effects of Mangosteen Pericarp Water Extract through Antioxidative Neuroprotection and Anti-Apoptotic Action
Source: Antioxidants (Basel). 2020 Dec 30;10(1):34. doi: 10.3390/antiox10010034 (PMC7823671; doi:10.3390/antiox10010034)

Figure S1: UPLC-UV chromatogram of mangosteen water extract at 254 nm wavelength

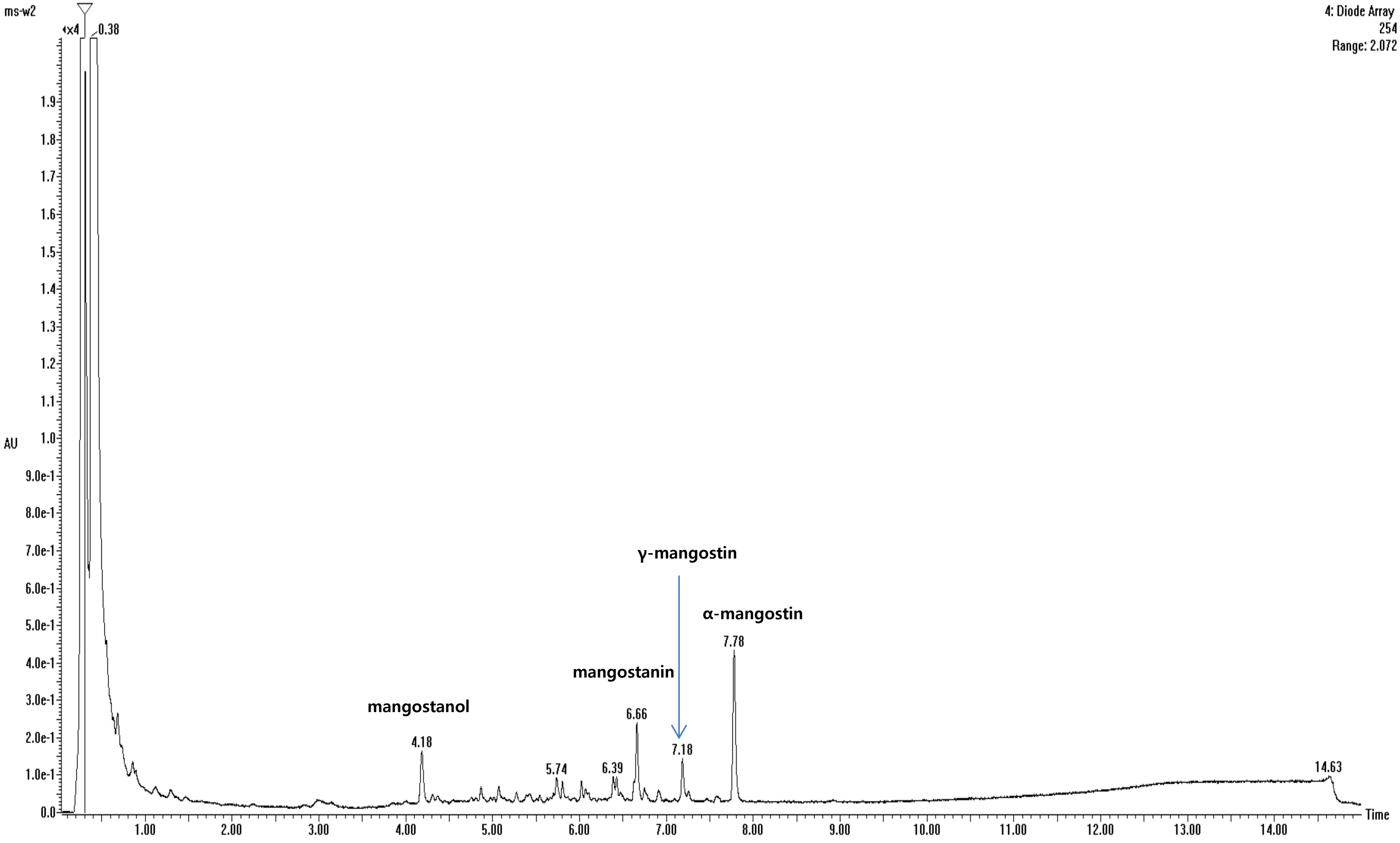

Figure S2: UPLC-UV chromatogram of mangosteen water extract at 270 nm wavelength

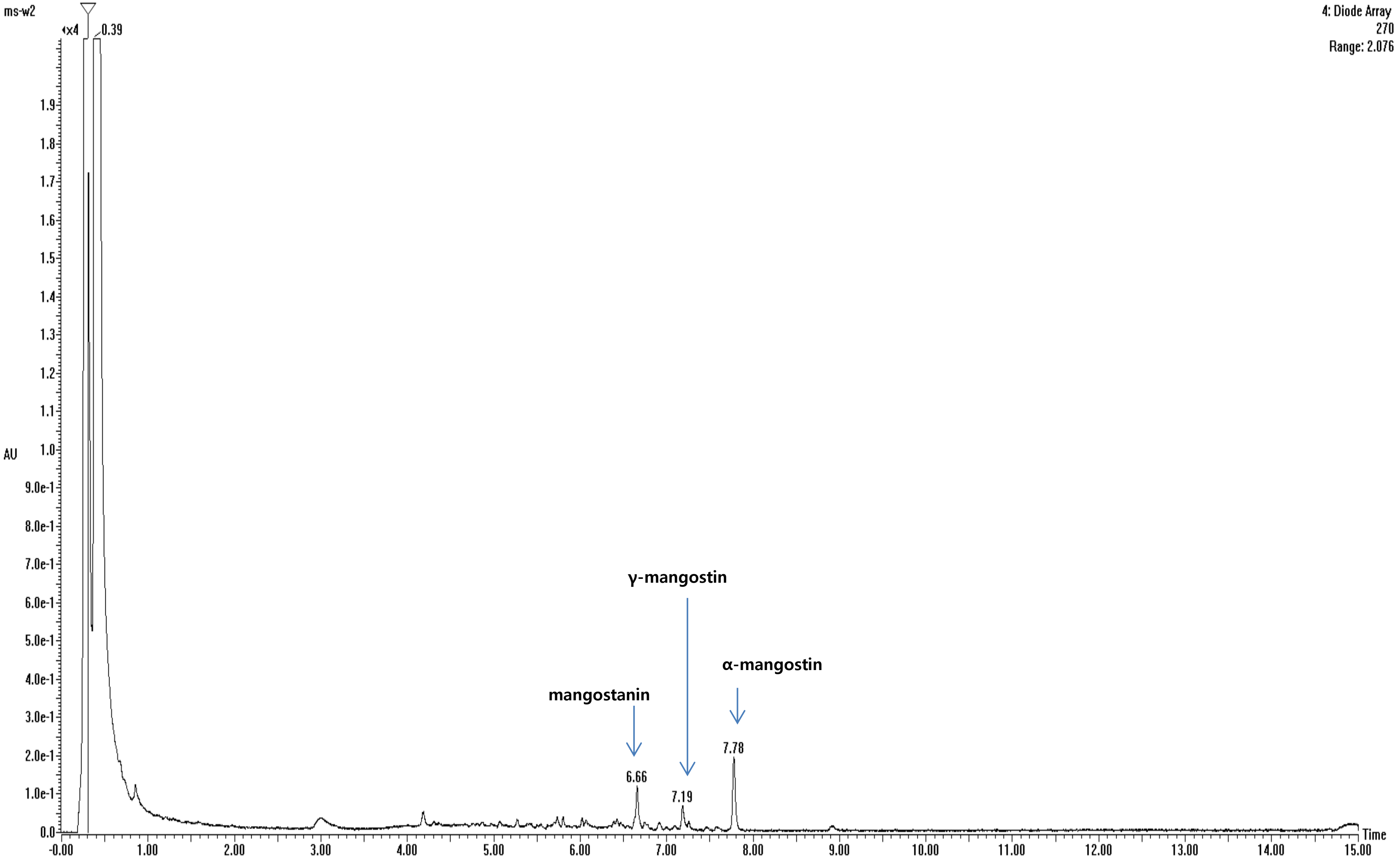

Figure S3: Mass chromatogram of mangosteen water extract

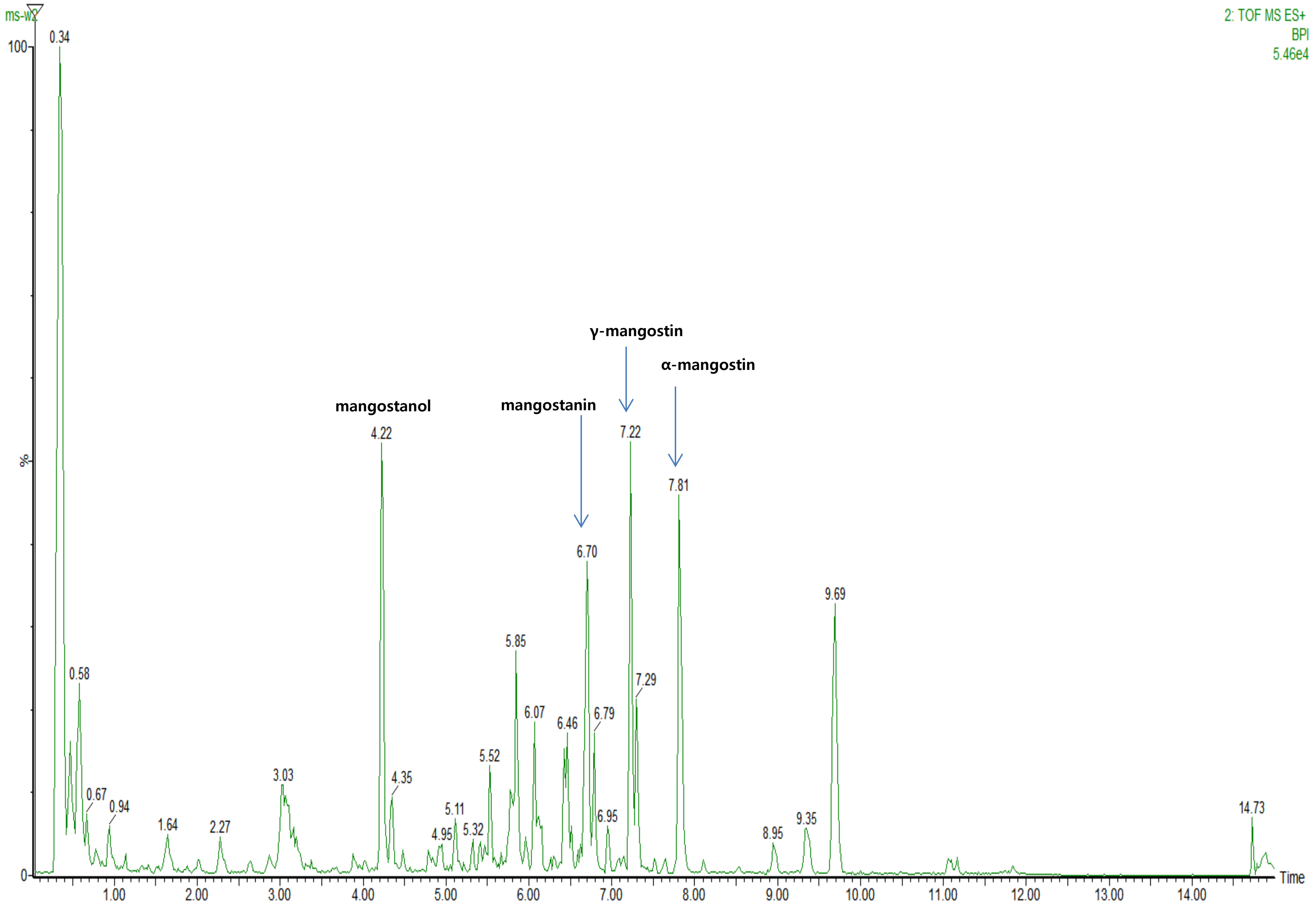

Figure S4: Mass spectrometric data of individual peaks at different retention time

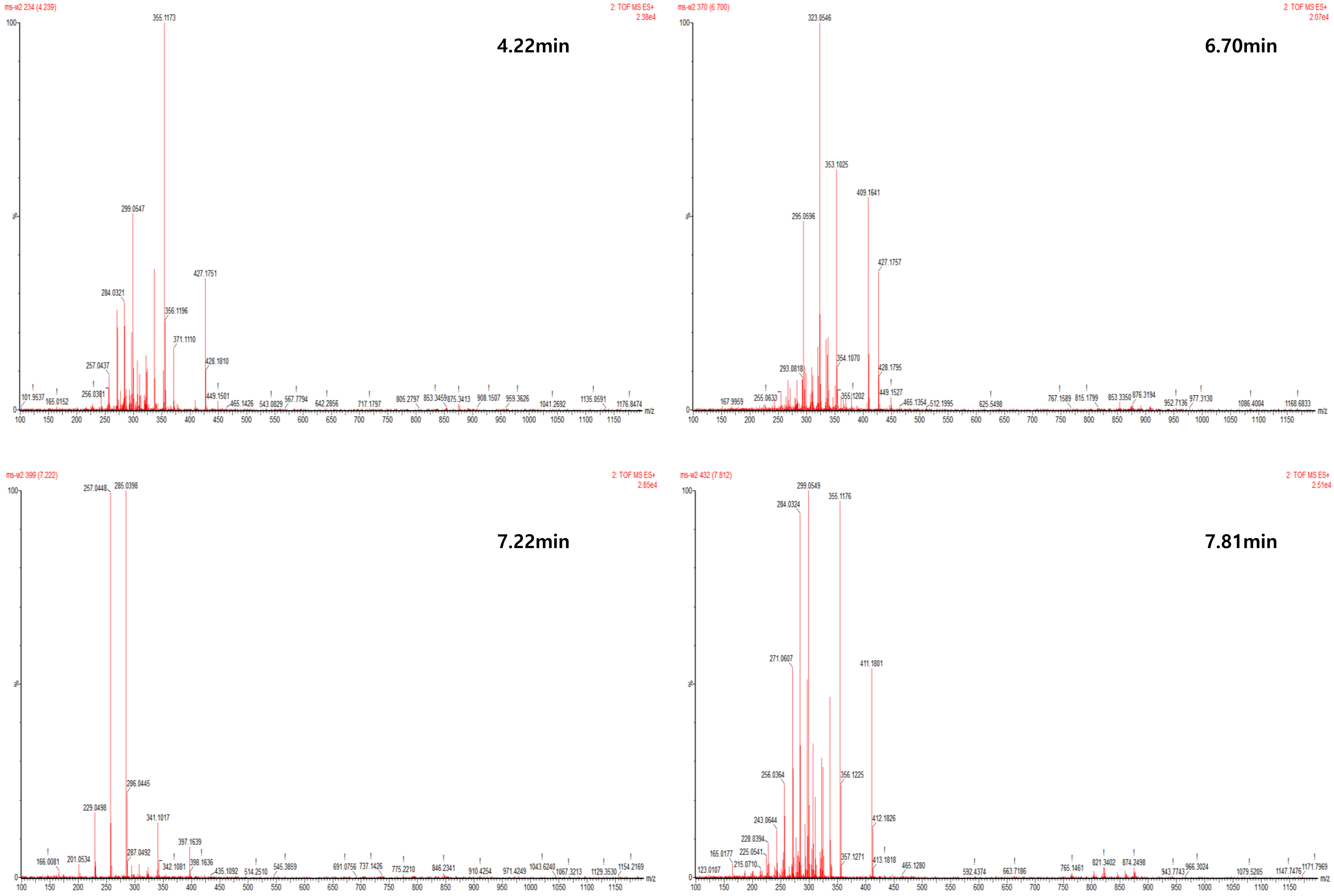

Supplement: Supplementary file 1 [file antioxidants-10-00034-s001.pdf]
